# Web pages: What can you see in a single fixation?

**DOI:** 10.1186/s41235-018-0099-2

**Published:** 2018-05-09

**Authors:** Ali Jahanian, Shaiyan Keshvari, Ruth Rosenholtz

**Affiliations:** Department: Computer Science and Artificial Intelligence Laboratory (CSAIL), Institution: Massachusetts Institute of Technology, Cambridge, MA USA

**Keywords:** Gist, At a glance, Web page gist, Layout, Ad, Web design, Visual perception

## Abstract

Research in human vision suggests that in a single fixation, humans can extract a significant amount of information from a natural scene, e.g. the semantic category, spatial layout, and object identities. This ability is useful, for example, for quickly determining location, navigating around obstacles, detecting threats, and guiding eye movements to gather more information. In this paper, we ask a new question: What can we see at a glance at a web page – an artificial yet complex “real world” stimulus? Is it possible to notice the type of website, or where the relevant elements are, with only a glimpse? We find that observers, fixating at the center of a web page shown for only 120 milliseconds, are well above chance at classifying the page into one of ten categories. Furthermore, this ability is supported in part by text that they can read at a glance. Users can also understand the spatial layout well enough to reliably localize the menu bar and to detect ads, even though the latter are often camouflaged among other graphical elements. We discuss the parallels between web page gist and scene gist, and the implications of our findings for both vision science and human-computer interaction.

## Significance statement

**Fig. 1:**
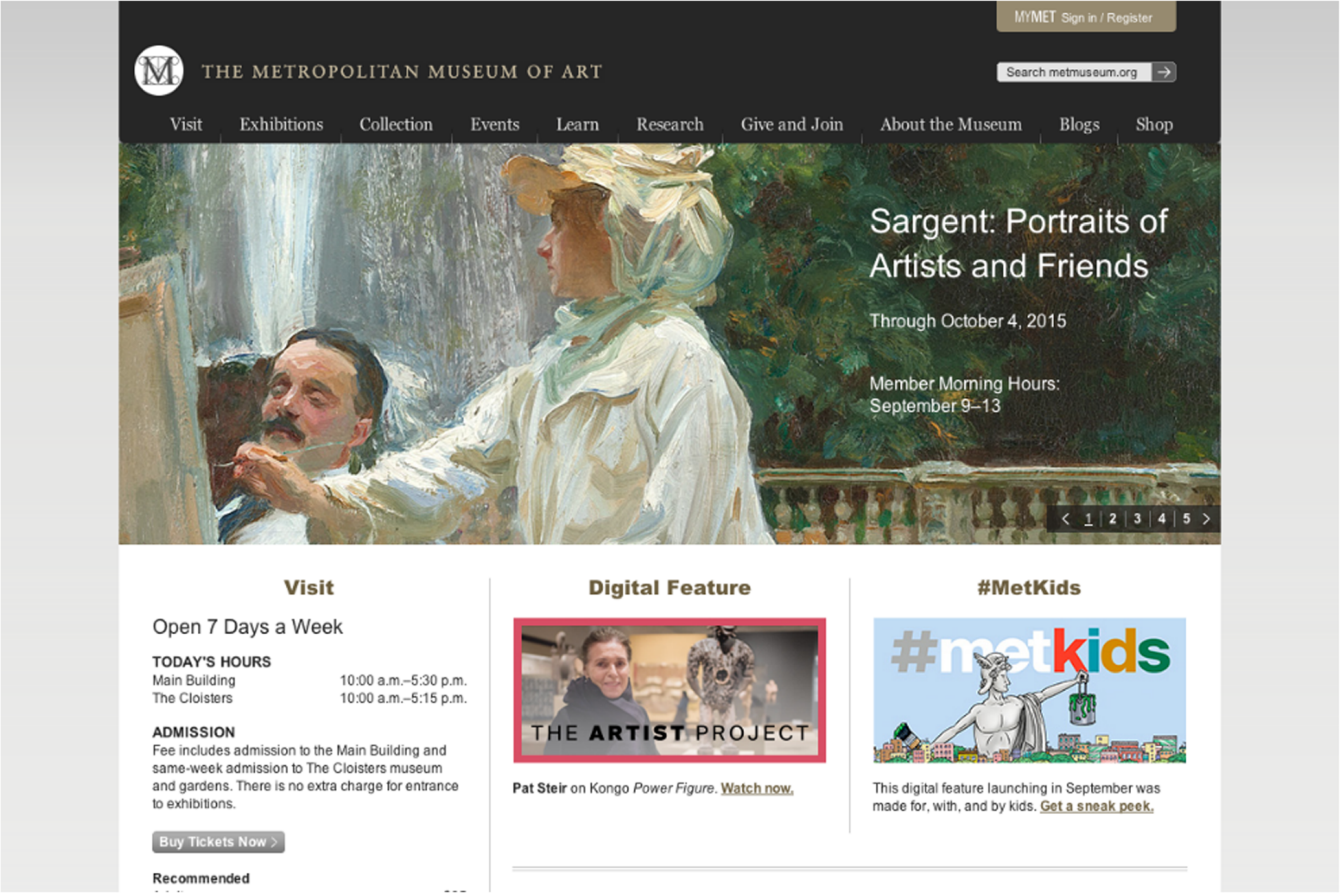
What can one see in a single glance at a web page? Is it obvious that the page belongs to an art museum? Can one tell the opening hours, or at least find where they are listed? What about finding the location of the navigation menu or the search box?

What a user can see at a glance at a web page affects their use of that page (e.g., see Fig. 1). For instance, while searching online, rapid assessment of web page category can facilitate judgments of whether that page likely answers the query. Designers strive to persuade users to stay on their web page instead of leaving it for another. If a user cannot get much information from a glance, this can detract from usability, by forcing the user to spend time understanding the page layout or assessing page content. This in turn could require reading a significant amount of the page’s text, a slow and perhaps frustrating process. What a user perceives at a glance also affects whether they are distracted by or click on an ad. At-a-glance perception of natural scenes has been extensively studied using tasks such as animal/no animal and judging scene category, informing both scene perception and basic science of vision mechanisms. However, those results do not obviously generalize to at-a-glance perception of designed, text-heavy web pages. We ran several in-lab studies, and found that subjects are above chance at perceiving the category of a web page in a single eye fixation. They can also reliably detect ads and localize the menu bar. While a complex design like a web page is unlikely to be fully comprehended at a glance, if it is well designed, it will contain adequate cues for a holistic understanding of the page and planning additional information foraging (Pirolli & Card, 1999).

## Background

A fundamental constraint on performance in any visual task is the information available at a glance. Searching for a person in a natural scene will be more difficult if we cannot detect them at a glance. Being able to at least extract target-relevant features at a glance will speed up search; otherwise, we may have to execute a number of eye movements to find the target. Our ability to quickly get the gist of a scene can also speed up search if it provides layout information, identifying likely locations to find a person.

In the context of navigating a web page (e.g., see Fig. 1), clearly much of the pertinent information comes in the form of text that may not be readable at a glance. However, if a user can quickly determine that a web page is a blog, and thus, say, an unreliable source of information about drug interactions, she can quickly navigate to another page. (Of course, in viewing a page of search results, a savvy user might also realize that a given link points to a blog based on its URL. However this cue might not always be so readily available.) To the extent that a user can determine the layout of the page at a glance, she can easily direct her attention to a paragraph of interest, click on a button relevant to her goals, or look through the menu for more choices. At the other extreme, if the user cannot get much information out of a page at a glance, she may be forced to read a significant amount of the text, or otherwise scan the page, a slow and perhaps frustrating process. The user may decide simply to navigate to a new page that is more easily comprehensible – an undesirable outcome from the point of view of the page designer, the owner of the page, and any companies with ads on that page. Understanding the information available at a glance constrains models of perception, visual representation, and attention, and informs our understanding of usability and design.

A considerable amount of information about a stimulus, such as a scene or display, is available in a single fixation. This summary information has been termed the “gist.” Colloquially, the gist is defined as “the sentence one uses to describe a stimulus.” Often this is operationalized as “the perceived contents of a scene given a certain amount of viewing time” (Fei-Fei, Iyer, Koch, & Perona, 2007), often in a single fixation (Fei-Fei et al., 2007; Oliva, 2005). We take the gist to mean the information available at a glance, i.e., in a single fixation. Such a fixation can last between 100 and 300 milliseconds (ms) (Harris, Hainline, Abramov, Lemerise, & Camenzuli, 1988; Pieters & Wedel, 2012; Wedel & Pieters, 2000), while typical fixations fall in the range of 200–250 ms (Rayner & Castelhano, 2007).

At a glance, participants can identify the category of a natural scene (e.g., beach vs. forest, indoor vs. outdoor, parking lot vs. downtown) (Ehinger & Rosenholtz, 2016; Greene & Oliva, 2009b; Joubert, Rousselet, Fize, & Fabre-Thorpe, 2007; Rousselet, Joubert, & Fabre-Thorpe, 2005), and how much room there is to navigate (Greene & Oliva, 2009a). They can determine whether a given object is present, such as an animal (Kirchner & Thorpe, 2006; Li, VanRullen, Koch, & Perona, 2002; Thorpe, Fize, & Marlot, 1996), vehicle (VanRullen & Thorpe, 2001), or a human face (Crouzet, Kirchner, & Thorpe, 2010). They can reliably distinguish between cities (e.g., Paris vs. Los Angeles) and tell what kind of intersection lies ahead (Ehinger & Rosenholtz, 2016). Furthermore, experiments in which participants freely report what they perceived in the scene, as opposed to merely carrying out a pre-defined task, have revealed the richness of the perception of lower and mid-level properties, such as the colors and textures present (Fei-Fei et al., 2007).

In addition to the extensive research on natural scenes, much of vision research has (effectively) studied vision at a glance using artificial, psychophysics-style stimuli (e.g., Gabors, simple 2D/3D shapes, synthetic textures, etc.). Many experiments studying basic visual abilities use short display times, typically only long enough for a single fixation. This includes studies of texture segmentation (Julesz, 1981; Rosenholtz & Wagemans, 2014; Treisman, 1985), popout search (Treisman & Sato, 1990), crowding (Levi, 2008), ensemble/set perception (Whitney, Haberman, & Sweeny, 2014), numerosity judgments (Feigenson, Dehaene, & Spelke, 2004), dual-task performance (VanRullen, Reddy, & Koch, 2004), iconic memory (Sperling, 1960), and perceptual organization in general (Wagemans, 2015). Experimenters use short display times not only to explicitly study at-a-glance perception; but also to study preattentive processing, or avoid complicating factors, such as fixation location. Human vision research, however, rarely extends this work to information visualizations, computer displays, and user interfaces; all of which have scene-like qualities and are practically relevant, despite being artificially designed. The goal of our research is to bridge this gap between natural and artificial stimuli by studying at a glance perception of web pages.

Research on human vision arguably suggests that perception of artificial stimuli is poorer than that of natural scenes. Synthetic stimuli and tasks appear to be more affected by attentional limitations than natural stimuli and tasks (Li et al., 2002). Researchers have suggested several explanations for this apparent difference. Our visual systems developed to process natural stimuli (Geisler, 2008). There appear to be brain areas devoted to processing stimuli like natural scenes (Epstein & Kanwisher, 1998); this specificity of neural organization possibly provides an advantage in processing those natural stimuli (VanRullen et al., 2004). In addition, web pages are often quite text-heavy; much of this text is unlikely to be readable at a glance, perhaps further impairing ability to classify a web page at a glance. One obviously cannot generalize from extracting the gist of a natural scene or of psychophysics-style stimuli to the gist of diversely designed artifacts such as web pages. Given their novelty and pervasiveness, web pages are “real-world” stimuli that require rigorous psychophysical investigation.

Beyond contributing to theories of human vision, understanding web page gist is relevant for design and usability. We can learn from web pages that are easy to comprehend at a glance in order to improve easy access to relevant information. For this reason, researchers in the HCI (Human-Computer Interaction) community have begun to study perception of web pages at a glance. However, to our knowledge all of these studies involved subjective judgments, e.g., “is this web page aesthetically pleasing,” or “does this web page *appear to have* high or low usability?” Researchers have found that participants form subjective impressions of the appeal of a web page in the first 50 ms of viewing, and respond consistently when shown the same stimulus later (Lindgaard, Fernandes, Dudek, & Brown, 2006). Furthermore, first impressions of visual appeal based on short (50 ms) exposures correlate well with judgments based upon longer viewing times (500 ms and further up to 10 s) (Tractinsky, Cokhavi, Kirschenbaum, & Sharfi, 2006). Users also make consistent subjective ratings about the trustworthiness and perceived usability of web pages after only 50 ms of viewing (Lindgaard, Dudek, Sen, Sumegi, & Noonan, 2011). Inspired by human vision research (see Oliva & Torralba, 2006) that suggests that low spatial frequencies are sufficient to communicate the layout of a natural scene, Thielsch & Hirschfeld (2010, 2012) found high correlation between judgments of aesthetics made on low-pass filtered web page screenshots and the original web page screenshots. Perceived usability, on the other hand, correlated better with judgments made based on high-pass filtered stimuli. Of course, just because observers can consistently make certain subjective judgments at a glance does not imply that they will be able to perform the tasks of interest in this paper. Instead of studying subjective judgments, we ask observers to perform objective tasks with web pages at a glance.

We perform several experiments to investigate what can be seen in a single fixation, 120 ms, on a web page. Display times of this magnitude are typical for similar studies with natural scenes (Fei-Fei et al., 2007). In Experiment 1, we ask whether observers can rapidly ascertain the category of a web page. This is a new question in the human vision literature. Common wisdom in HCI suggests that a user cannot acquire much semantic information, such as the category of a web page or meaning of any text, in a presentation time of less than 500 ms (e.g., Lindgaard et al., 2006). However, researchers have not actually tested this hypothesis.

In Experiment 2, we ask whether ads are detectable at a glance. This is an object detection task like the animal/no-animal task in scene perception studies (Kirchner & Thorpe, 2006; Li et al., 2002; Thorpe et al., 1996). However, since detection depends greatly on both the signal to be detected and on the background against which it appears, one cannot infer from easy animal detection that ads will be easy to detect. In particular, designers may use multiple different strategies for ad design. Some designs aim for ads to have a salient, visually distinct appearance from the rest of the web page, while other designs might disguise the ad on purpose, effectively creating camouflaged objects. In previous work, Pieters & Wedel, (2012) showed that observers can distinguish between ads and editorial articles in magazines with high accuracy (up to 85% on average) in only 100 ms. Furthermore, observers could discriminate between types of ads (i.e., for cars, financial services, food, or skincare products), at rates of 95% correct for “typical” ads, and 53% correct for “atypical” ads. This study differs from the present in several ways. We study ads embedded in web pages, as opposed to isolated full-page magazine ads. The task is to detect these embedded ads, rather than to categorize them. In addition, the ad style in magazines tends to be quite different from that in web pages.

Finally, in Experiment 3, we ask how well a user can locate the menu bar. A menu bar is essentially defined by the horizontal or vertical alignment of its elements; menu items form either a row or a column, respectively (Fig. 10). In addition, many menu bars contain menu items that have similar colors and other features, and/or those items may be contained within a rectangular box. As a result, one can think of menu localization as an initial question of what perceptual organization (alignment, similarity, and/or containment) one can perceive at a glance at a web page. Considerable work has demonstrated that observers can perform perceptual organization tasks in brief presentations (van der Helm, 2014). However, much of this work uses fairly simple and homogeneous displays, leaving open the question of what observers can perceive in web pages at a glance. Perhaps more relevant is work suggesting that observers can estimate the 3D layout of a natural scene at a glance (Greene & Oliva, 2009a, 2009b), though clearly both the task and stimuli differ greatly from detecting a web page menu.

Our particular set of tasks can be thought of as a parallel to tasks in the scene gist literature. We have a semantic task (categorization), similar to scene categorization (Biederman, 1981); an object detection task (ad detection), similar to object detection with scenes (Thorpe et al., 1996), and a layout-related task (menu localization), similar to 3D layout estimation (Oliva & Torralba, 2001). Can observers assess these mid-to-high level properties in web pages, as they can in natural scenes?

## General experimental procedure

Observers viewed stimuli on a Dell E2209W LCD monitor (47.5 cm by 30 cm viewable area, 1680 × 1050 resolution) with their eyes approximately 55 cm from the center of the screen. Stimulus presentation and response collection was done using PsychToolbox-3 (Kleiner et al., 2007) in MATLAB.

In all experiments, observers responded by using the mouse to click on-screen buttons, and received no feedback. The order of presentation was randomized for each observer. Stimuli were screen shots of web pages, or modified versions thereof, as described below. Stimuli were displayed at their original 1200 × 800 resolution, and subtended an area of the screen approximately 33.75 cm × 22.5 cm (34 × 23 degrees visual angle). In other words, all web pages were displayed at the same size and resolution used to capture the screenshot, and viewed at a typical viewing distance for browsing the web. Nearly all of the text in the original screenshots was legible to any observer with normal or corrected-to-normal vision at the experimental viewing distance, with the only exceptions being (rarely) poorly rendered text, or essentially “footnote” or other unimportant text – e.g., disclaimers or trademark symbols.

### Participants

As web pages are designed artifacts, the appearance of a given category is at least in part a cultural convention (Reinecke, Arbor, & Gajos, 2014). As a result, performance will likely depend upon a user’s experience with web browsing, and with whether the user comes from the same culture as the designs studied (e.g., Indian news sites might look different from American ones). While cultural peculiarities are certainly an interesting avenue of study, they are not immediately relevant to our research goal. To minimize this potential added source of variance, all observers were university students (undergraduate and graduate), with English as their first language. In total (for all experiments), we recruited 25 participants (average age = 23.56 years, standard deviation = 4.00 years, range of 18 to 35 years, 12 female). The participants, on average, spent 3 h per day surfing the Internet. Each experiment took each participant between 30 min and an hour to complete, and participants received $15 USD compensation for their time. All participants provided written informed consent prior to the experiment.

## What are the web page categories?

In order to rigorously study at-a-glance web page categorization, we need properly labeled web pages. One way to get labels is to do a categorization experiment with unlimited viewing time. In other words, we need to collect a corpus of web pages, define a set of unambiguous category labels, and confirm that observers agree on the ground truth labeling of those web pages’ categories.

### Picking the web page categories

Our goal was to divide web pages into “common-sense” categories which would feel natural to our participant population. For example, common-sense categories used for scene perception research include “beach,” “park,” and “office;” likewise, we refer to “company web page,” “blog,” and “online shopping site.” We conceived of categories in terms of the use of the web page. Users go to a news page to learn about current events, to a tourism page to learn about a travel destination, etc. Out of a larger set of common web page categories, we chose a subset that minimized overlap in their definitions. It would be confusing, for instance, to allow both “sports” and “news” as categories, as many news web pages might report sports news, making the category of the page ambiguous. Table 1 lists the resulting categories and the definitions provided to experimental subjects, which they could view at any time during the experiment.

**Table 1:** Selected web page categories and their descriptions. This is identical to the list that was given to subjects, and was designed to be intuitive. Each subject could consult this list at any time during the experiment

Category	# of screenshots	Description
1. Art place	30	if you want to see art, e.g., exhibition, museum, galleries
2. Blog	32	has articles with titles and dates, it usually has opinion of a person with a form that you could write your comments in
3. Company	33	if you want to learn about the services that it provides, e.g,. consultation
4. Computer game	33	if you want to play a computer game either online or offline, or read the latest news about a computer game, buy or download a computer game
5. Helpline	32	if you have an emergency, e.g., need advice for your kid
6. News	34	if you want to read daily news
7. Online tutorials	28	e.g., learning a course, finding a tutorial for learning html coding
8. Shopping	34	if you want to buy an online product item, new or used
9. Society	37	unions, groups of people with same interests, e.g., if you are looking for a cultural club, or book reading club
10. Tourism	41	if you are looking for things to do in a destination, booking for a tour

### Collecting web page screenshots

In order to gather a candidate set of web page stimuli, we first collected web pages belonging to the 10 categories. One way to do this would have been to search for keywords (e.g., “news,” “art museum”) in an online search engine. However, we did not want to bias our set of web pages to those that come up first on a web search. In particular, we wished to avoid selecting high-traffic web pages, as these might lead to anomalous responses due to familiarity with the logos, color scheme, or layout of those particular web sites. Thus, rather than using search engines to find category exemplars, we crawled the site DMOZ.org for 1795 random URLs from our selected categories. DMOZ is a widely used online repository of URLs, organized by volunteer editors into categories and sub-categories. The unbiased collection of URLs is reflected by its use in professional web page traffic-ranking services like Alexa (alexa.com) and as a database for training URL-based topic classification algorithms (Baykan, Henzinger, Marian, & Weber, 2011). We automatically captured web page screenshots in a 1200 × 800 pixel browser window using the webkit2png software package (https://github.com/adamn/python-webkit2png) in the Safari browser on an Apple desktop. We only kept the first “page,” i.e., the portion of the web page that fit in the browser; thus, some screenshots did not span the full vertical extent of the web page. Given that the first glance of a web page occurs before users can scroll further down the web page, and that these browser dimensions are typical for current display resolutions, our screenshots provide a good representation of the first 120 ms of the typical web browsing experience. By hand, we culled stimuli that appeared, upon examination, to be ambiguous in category, written in a language other than English, or not fully loaded at the time of screenshot. This narrowed our initial set of screenshots to 714.

### Getting ground truth categories for screenshots

In order to more objectively assess whether observers would agree with the labeled category for each screenshot, given unlimited viewing time, we asked 6 participants in a pilot experiment to categorize each screenshot into one of the 10 categories. Of the initial 714 screenshots, we randomly selected a subset of 379, such that the experiment took approximately one hour. Each participant categorized all 379 screenshots. Presentation order was randomized across participants.

Half of the participants were male, and all were native English-speaking university students. We discarded the data from one observer because of apparent difficulty understanding the instructions. A row of response buttons, one for each category, appeared below each screenshots. Although participants were allowed unlimited viewing time, they were encouraged not to spend more than several seconds on any particular web page. This was to avoid deciding upon the category based on small or technical details in the text; we wanted to avoid having participants overthink the categorizations, such as deciding that a blog is a tourism web page because many of the posts involve travel.

Prior to the experiment, we gave the observers a verbal overview of each category. The participants were also instructed to consult with a list of all 10 categories and their short descriptions (see Table 1), while making their decisions. This list was displayed on a separate screen. The same was true for the at-a-glance categorization experiments described below.

For 283 web pages (about 75%), all 5 observers agreed with our categorization, and for 334 (about 88%) at least 4 of 5 agreed. In further experiments, we use only web pages for which at least 4 out of these 5 observers agreed with our initial categorization. This set of web pages has the following number of exemplars per category: art place, 30; blog, 32; company, 33; computer game, 33; helpline, 32; news, 34; online tutorial, 28; shopping, 34; society, 37; and tourism, 41. For some examples of these stimuli, see Fig. 4.

## Experiment 1A: Can observers categorize web pages at a glance?

### Participants

We recruited 10 participants for this task (5 female).

### Procedure

Each trial consisted of three steps. First, participants were asked to fixate on a cross that appeared on the screen for 900 ms. We asked participants to fixate the center of the page, consistent with many previous scene perception experiments. For web pages, previous work has examined where people look while surfing the Internet, and found that for information foraging tasks (e.g., “which car has the best performance, a Porsche, BMW, or Audi?,” subjects mainly look at the center of the web pages in the first second of viewing (Buscher, Cutrell, & Morris, 2009). Future work could examine web page perception when fixating at typical fixations during a particular task.

**Fig. 2:**
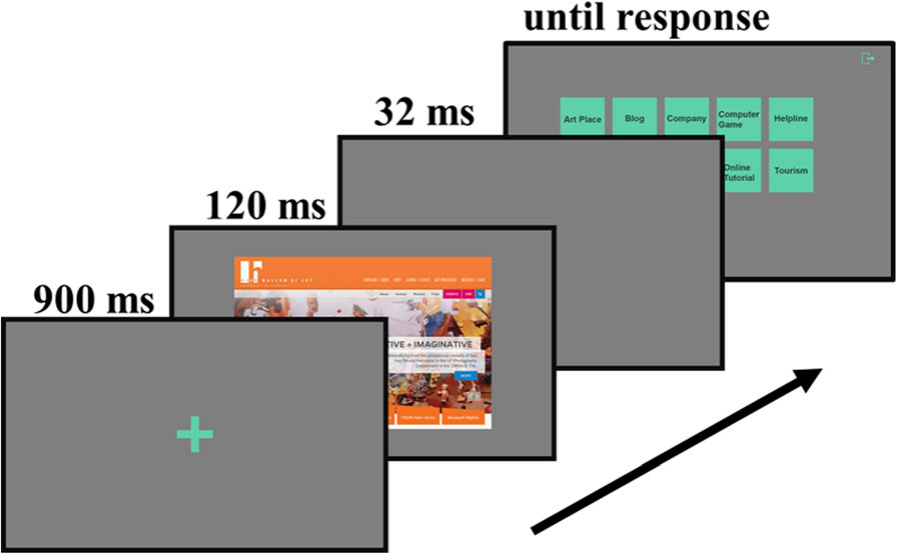
Schematic of the gist experiment. Participants were instructed to fixate on a cross in the center of the screen. A screenshot appeared for 120 ms, then was replaced with a response screen after a short blank screen. Participants had unlimited time to make their response, then the next trial began

The fixation cross was then immediately replaced by a screenshot of a web page. After 120 ms, the screenshot was removed. The response buttons appeared 32 ms later, and remained until the participant made a response. The buttons then disappeared, and the fixation cross for the next trial appeared after 250 ms (see Figs. 1 and 2). Because the response screen appeared almost immediately after the web page, and the response was a relatively structured screen, we did not add an additional mask screen after the web page presentation. We, however, address this potential issue in Experiment 1C. Each participant categorized 379 web pages, of which we analyzed results for the 334 selected for consistent categorization in unlimited viewing conditions (see the online supplement https://github.com/ali-design/WebpageGist for all experimental stimuli).

Participants first did a short training session of 30 trials to orient them to the experiment. The screenshots used in the training came from a separate set than used in the main experiment.

### Results

**Fig. 3:**
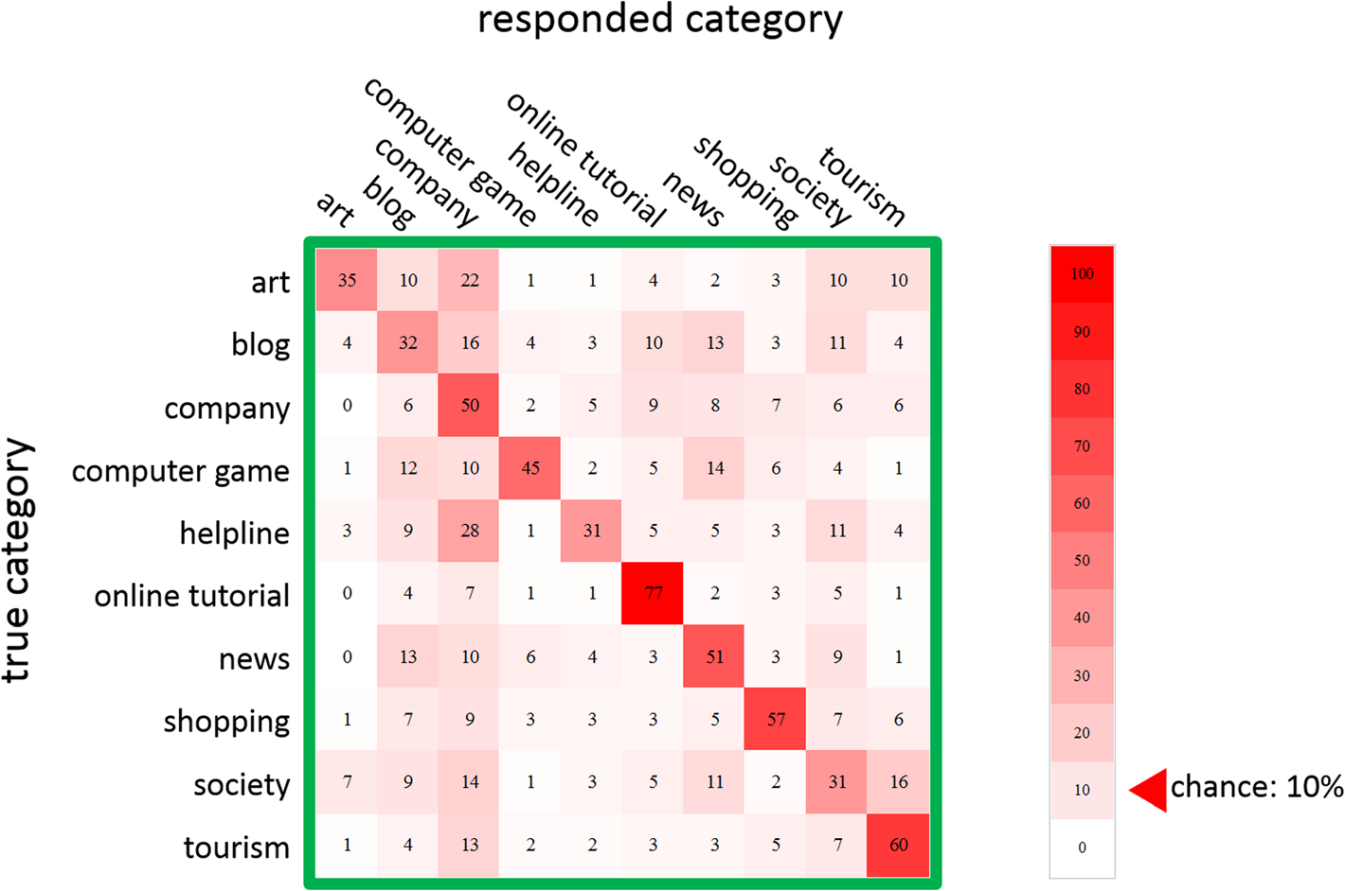
Results of the gist experiment. Each row denotes the correct category of a given figure, and each column the responded category. The value in each cell is the percentage of trials which, for each true category (row label), the screenshot was identified as the category given by the column label. Values along the diagonal indicate the percentage of correct responses per category

**Fig. 4:**
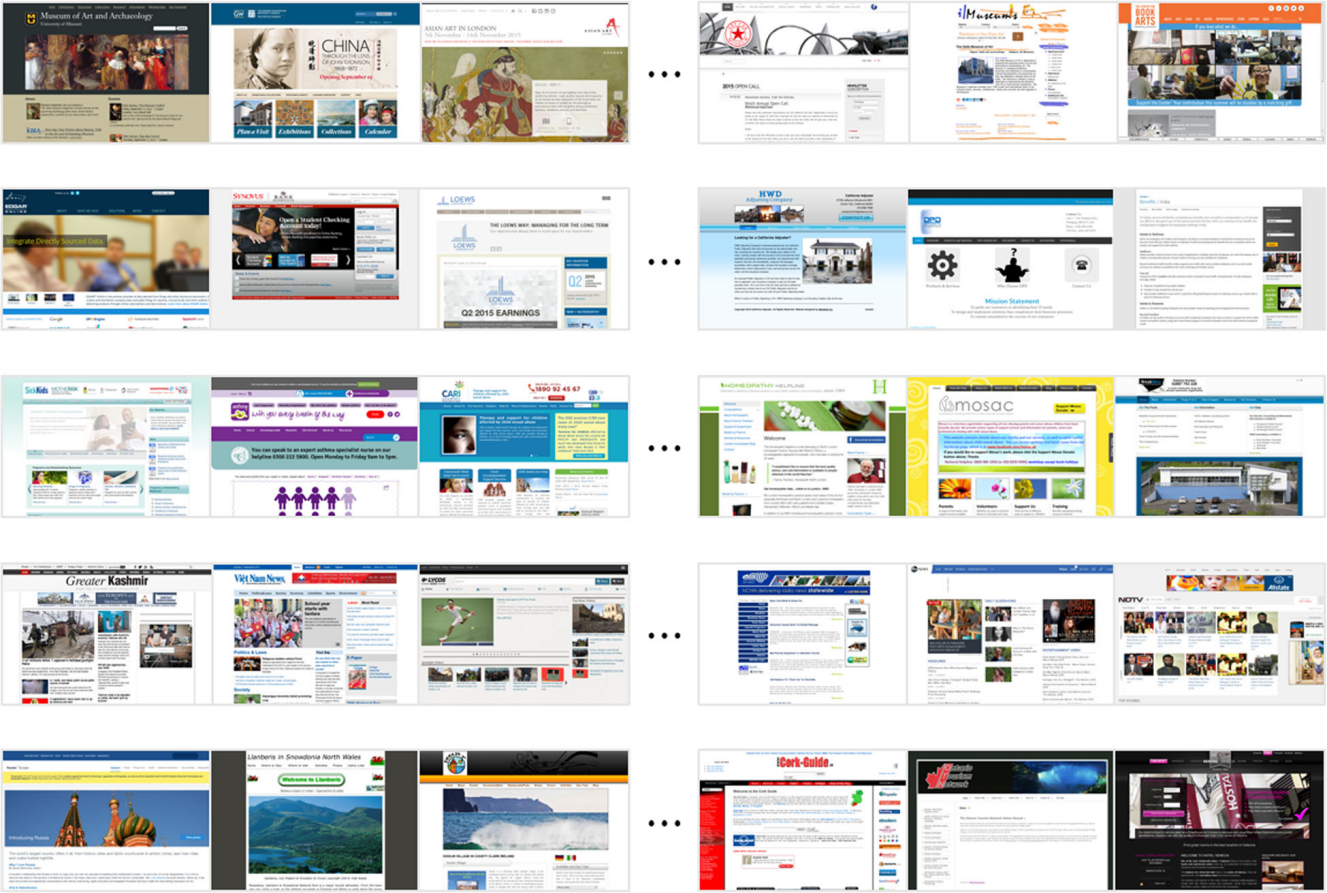
Example web pages from our corpus. Each row corresponds to a different web page category: (row 1) art place; (row 2) company; (row 3) helpline; (row 4) news; (row 5) tourism. The first three web pages in each row have the highest performance over all subjects, while the last three have the lowest performance, for each category

Unless otherwise noted, all tests are Bonferroni-corrected two-sided permutation tests. We analyze the data rigorously using randomized, nonparametric permutation tests rather than traditional hypothesis tests, such as *t*- or *F*-tests, since categorization tasks do not meet the required underlying normality assumptions (Still & White, 1981). We aggregated responses from all 10 participants and computed confusion matrices (Fig. 3). Averaged over all subjects, performance was well above chance (*M* = 47% correct, *SD* = 4.5 percentage points (pp), *p* < 1E-5, chance performance = 10% correct) and each subject’s performance individually was well above chance (*p* < 1E-5, chance = 10%). We use percentage points to describe standard deviation over percentages, e.g., 47% +/− 4.5 pp. refers to the range from 42.5% to 51.5%. Furthermore, performance in each category was also well above chance (*p* < 1E-5, chance = 10%) when averaged across subjects (average performance per category are the diagonal elements in Fig. 3). Mean and standard deviation of performance per category, computed over subjects can be found in Table 2. For samples of the best and worst web pages with respect to performance, see Fig. 4. Since we have between-subjects standard deviations for each of 10 categories as well as 90 different confusions, we report them in the online supplemental information (https://github.com/ali-design/WebpageGist).

**Table 2:** Mean (in percent correct, top value in each cell) and standard deviation (in pp., bottom value in each cell) of performance in experiments 1A and 1B, computed across subjects (*N* = 10 per experiment) per category. The mean values are identical to the diagonals of the confusion matrices in Figs. 3 and 6

	Art	Blog	Company	Computer game	Helpline	Online tutorial	News	Shopping	Society	Tourism
Intact text (Exp. 1A)	*M* = 35	32	50	45	31	77	51	57	31	60
	*SD* = 10	12	16	11	13	10	10	9	20	10
Scrambled text (Exp. 1B)	31	39	35	50	34	73	29	55	20	60
	13	12	9	14	12	10	18	5	7	11

What cues in the stimuli might support such high performance? Interestingly, several participants indicated to us that they could read words within the screenshots, and that they used this information in doing the categorization task. We explored this possibility in Experiment 1B.

## Experiment 1B: Gist of scrambled web pages

Was some of the web page text at least partially readable? Did reading text help observers determine the category? A common assumption in HCI design is that text is not readable at a glance (Lindgaard et al., 2006). Research in human vision, however, suggests that participants could plausibly have read some of the text. Humans can read at least 12 words per second (83.3 ms per word) when presented foveally, one after another (Potter, 1984). Humans can also read words presented peripherally for short times, provided that both the font and the spacing between letters is large enough (Latham & Whitaker, 1996). If the spacing is insufficient, a phenomenon known as crowding strongly limits peripheral reading (Pelli & Tillman, 2008), and limits it far more than a lack of acuity (Rosenholtz, 2016). In addition, the participants need not literally *read* the text for it to be useful; they need only infer that words look more like words that would appear on, say, an art site than on a helpline page.

In order to test the hypothesis that observers are using text to classify web pages at a glance, we changed the text on a set of web pages to be unreadable. We then measured to what degree categorization performance degraded. If observers do not read any text at a glance, performance should be unaffected. On the other hand, if participants can read some of the text, there should be a significant effect of performance.

### Scrambling web page text

Previous work has used a number of techniques to render text unreadable, e.g., on a web page, including “Greeking” text (Tullis, 1998) or converting it into a language that our participant population is unable to read, such as *Finnish*. We chose instead to use image operations to flip the text about a horizontal axis, i.e., flip (using minimal bounding boxes around words). This enabled us to change all text, including text embedded in images, while minimally affecting font and layout of the page.

**Fig. 5:**
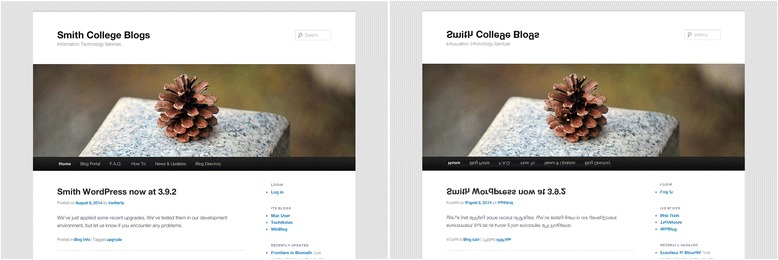
Screenshot of a blog web page (left), and the text-scrambled version (right)

We used custom software developed by two undergraduate students (https://projectnaptha.com/) that uses the Stroke Width Transform (Epshtein, Eyal, Yonatan, Ofek, & Wexler, 2010) and connected-components analysis to detect letters in an image, and then vertically flips the bounding box for each letter. While very accurate, this process did miss some of the text, and occasionally flipped regions with no text. An author manually checked each scrambled screenshot, and inverted any text that was still readable, using image-editing software to manually select the minimal bounding box, and vertically flip it. The experimenter also restored any significantly large image regions that were mistakenly flipped by the algorithm. See Fig. 5 for an example of a screenshot with scrambled text. One can see that some text, such as the word “College” remains fairly readable even with this manipulation; this experiment should, if anything, underestimate the degree to which participants can use text to categorize a page at a glance.

### Participants

We recruited 10 new participants (5 female) for this task.

### Procedure

The procedure was identical to that in Experiment 1A, except that all of the text in each screenshot was scrambled (except for the initial training, which included 15 trials with scrambled screenshots as well as 15 trials with unmodified screenshots).

### Results

**Fig. 6:**
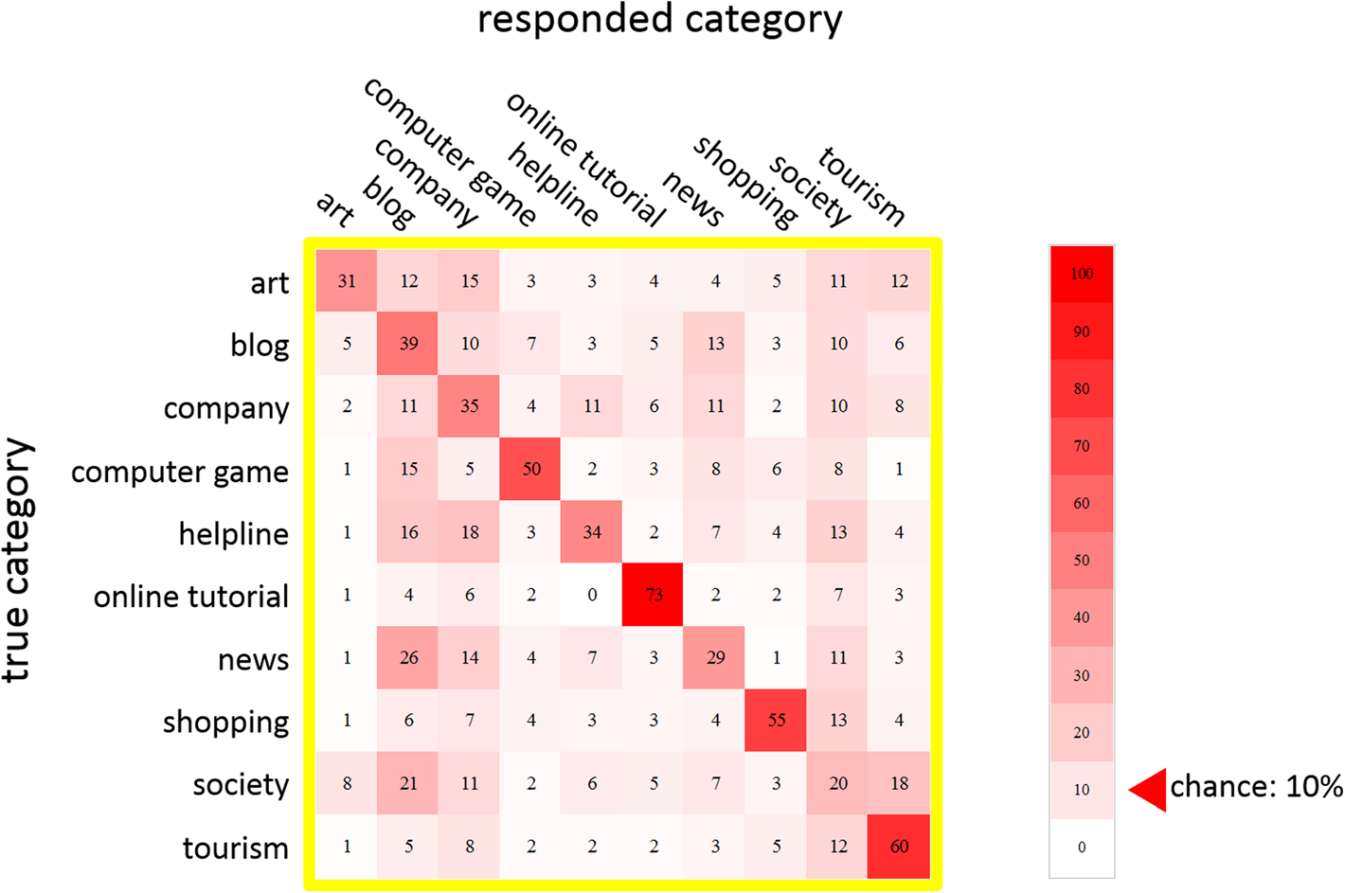
Results of the gist experiment with scrambled text. As in the previous confusion matrix, the value in each cell is the percentage of trials for which, for each true category (row label), the screenshot was labeled as indicated by the column label

As in Experiment 1A, we aggregated the responses from all participants and computed confusion matrices (Fig. 6). As with intact (unscrambled) web pages, performance averaged over subject was well above chance (*M* = 43%, *SD* = 3.7 pp., *p* < 1E-5, chance = 10%). Each subject’s performance was well above chance (*p* < 1E-5 per subject, chance = 10%). Furthermore, performance in each category was also well above chance (*p* < 1E-5 per category, chance = 10%) when averaged across subjects. The mean and standard deviation of performance per category (in units of pp) over subjects is reported in Table 2.

Importantly, we found that scrambling the text significantly decreased performance by 4.34% (*p* < 4E-4, collapsing over all subjects and categories); the categorization task is more difficult when the text is unreadable. This implies that participants in Experiment 1A were in fact inferring the category in part from readable text. Since performance in the scrambled condition was well above chance, however, readable text was clearly not the only cue. Further examining the individual web page categories, we found that performance was significantly better with intact than scrambled text for three categories: company (15.5 pp), news (22.14 pp), and society (11.62 pp) (*p* < 5E-3 per category, see Table 2 for standard deviation). Thus, readable text provided a better cue for some categories than for others; we examine this finding in more detail in the discussion.

## Experiment 1C: The effect of visual masking on categorization

It is possible that some visual processing occurs after the stimulus is removed, due to afterimages or iconic memory, despite the appearance of the response screen. This would effectively increase the stimulus display time, beyond the length of a typical fixation. To control for this possibility, we ran an experiment identical to Experiment 1A, except that immediately after each web page image, a noise mask appeared. The mask was made by taking a random web page from the unused subset of the original 714, transforming the image into the Fourier domain, randomizing the phase, and using the inverse Fourier transform to convert back to the image domain. The mask thus had the same spatial frequency content and color distribution as a real web page, but random phase.

### Participants

We recruited 7 new participants (4 female) for this experiment.

### Procedure

The procedure was identical to Experiment 1A, except that a mask appeared on the screen for 120 ms after the stimulus in the same position as the web page was on the screen.

### Results

All 7 observers performed above chance in all categories (*p* < 1E-5). Furthermore, there was no significant decrease in overall performance between the performance in this experiment than Experiment 1A (overall performance was 2.05 pp. lower in the masked experiment, *p* < 0.152), and there were no significant differences in performance for any categories. These results indicate that performance was not significantly affected by the mask, which presumably halts visual processing after 120 ms.

## Experiment 2: Does this web page have an ad?

In addition to perceiving the category of a real world scene, observers can also extract sufficient information at a glance to recognize a few objects (Fei-Fei et al., 2007; Oliva, 2005). Can participants also recognize elements of a web page at a glance? Advertising is one interesting and ubiquitous type of web content. Web pages often display ads to generate revenue, and advertisers in many cases want users to click on these ads. As a result, designers of both web pages and ads take care in setting the location and style of an ad to maximize the chances that a visitor will click on the ad. Users, on the other hand, may desire not to click on irrelevant ads, or perhaps learn to ignore ads completely. This might lead advertisers to use various techniques to “trick” users into clicking on the ad, for example when advertisers try to combat “banner blindness” (Benway, 1997).

In Experiment 2, we ask whether participants could detect the presence of an ad embedded in a web page at a glance.

## Participants

The same 10 participants in Experiment 1B completed this experiment in a separate session.

### Procedu**r**e

From our original set of 714 screenshots, we excluded 334 of the stimuli from the previous experiments. We then manually selected the first 50 with at least one visible ad, and the first 50 without. To qualify as having an ad, the screenshot had to display the entire ad, fully loaded, and the ad could not link to the same web site as the current page. Thus, we excluded ads for a different product sold on the same shopping site, e.g., a bank web page containing an ad about its own banking service. Overall, the experimental design was similar to Experiment 1A, except that the response screen had only two buttons, (one green for “ad,” one red for “no ad”), underneath the text, “Did you see any ad(s)?”

Participants did this task randomly interleaved with the one in Experiment 3 (finding navigational menus). After displaying each web page screenshot, the screen displayed (with equal probability) one of two questions: “Was an ad was present?” or “Where was the menu?” The participant answered only a single question per trial. The participant did not know which question would be asked until after the screenshot was removed from the screen. We made the task uncertain for two main reasons. First, this uncertainty better mimics standard web page viewing conditions. A typical user does not first approach a web page with the sole intention of finding any ads or isolating the menu; rather he has a higher level goal, which might at some point require locating a particular graphical element. While having two possible tasks is not identical to natural conditions, it is a step in that direction. Furthermore, we wanted to avoid participants deploying covert spatial attention or making eye movements to, say, a probable menu location, which would be more likely if the task were known. To put it more directly: an observer could simply look at the top of the web page and easily detect the presence of the menu, responding “top” if present and “left” if not present. Using our method, we can better test whether ads and menus are normally available at a glance when fixating the center of a web page, instead of testing how well observers can deploy overt or covert attentional mechanisms.

### Results

**Fig. 7:**
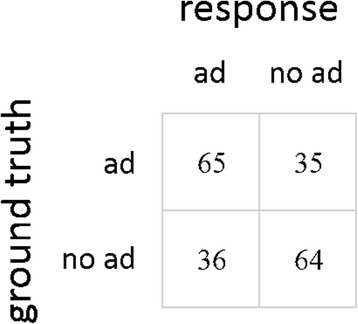
Results of the ad-detection task, in percent of responses. Diagonal values indicate correct classifications

The results are shown in Fig. 7. The row indicates the ground truth, and the column represents the response. Thus, for example, the top right quadrant indicates missed ads. Participants responded “ad” and “no ad” with roughly equal frequency. 8 of the 10 participants performed above chance (*p <* 0.03, chance = 50%), and the average performance collapsing over all subjects (*M* = 64.7%, *SD* = 7.4) was also well above chance (*p* < 1E-5). Thus, participants usually noticed ads within a single glance, much as previous studies have suggested that participants can easily notice an animal in a natural scene (Thorpe, Gegenfurtner, Fabre-Thorpe, & Bülthoff, 2001).

**Fig. 8:**
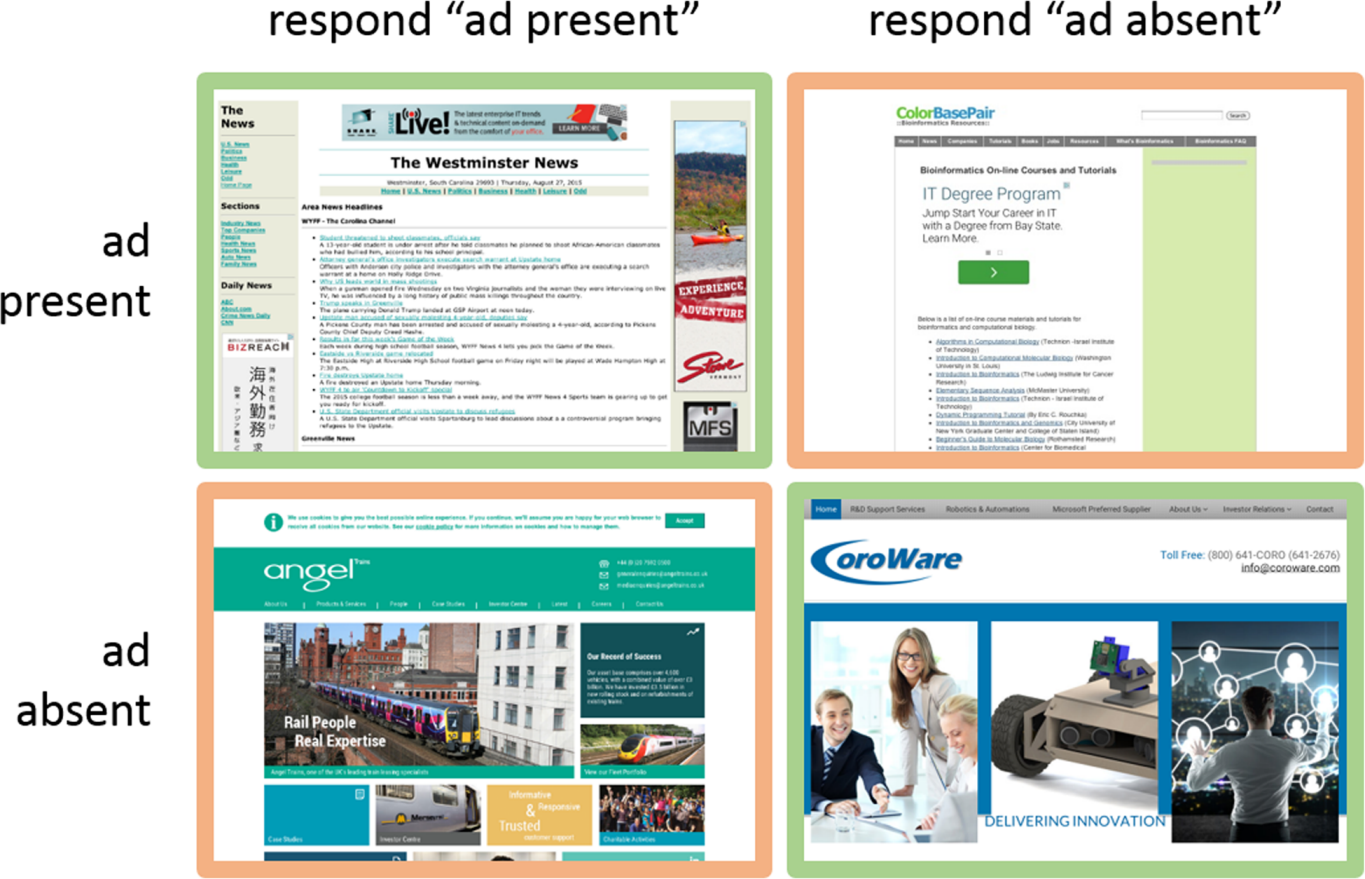
Examples of ad-detection stimuli. The top row web pages have an ad while on the second row web pages do not. The first column denotes web pages for which observers overwhelmingly responded “ad present,” while the second denotes predominantly “ad absent” responses. Therefore, the green outline indicates largely correct responses while orange indicates mostly incorrect responses

It is informative to examine some examples of correctly and incorrectly identified ad-present and ad-absent web pages from Experiment 2. The top right example in Fig. 8, for which only 1 of 10 participants detected the ad, has an ad that may be disguised as a style element. It is a text-based ad, where the text formatting is similar in style to that of the main content of the page. This is in contrast with the page in top left of Fig. 8 where ad is separate from the other visual groups on the page. Interestingly, in the bottom left example, 7 out of 10 readily respond “ad present,” even though an ad is not present.

## Experiment 3: Where is the menu?

A natural question to ask is whether users perceive the layout of a web page at a glance. For example, can they perceive columns and rows of text, and how different components are grouped? Can they tell apart interactive elements from purely aesthetic or informational ones? It is important to study perception of layout in web pages, because layout is an important factor in determining usability (Palmer, 2002).

Menus are vital for the effective use of a web page; beyond displaying the web page’s organization, the location of the menu suggests where to start looking for information on a page, and the status of the menu (e.g., the active item vs inactive items) helps the user place the content of a web page into context. For instance, an active menu item may indicate to the user that the visible content is what the user should focus on. Users’ fixations are often directed to the menu of a web page (Shrestha, Lenz, Chaparro, & Owens, 2007). Moreover, a menu’s content can suggest the purpose of the web page.

A designer can explicitly segment the menu from the rest of the page by using a different colored box, or implicitly by using other Gestalt grouping techniques. A menu bar is essentially defined by the alignment – either horizontal or vertical – of its elements (see Fig. 10). Thus, one can think of menu localization as one aspect of perceptual organization done at a glance with web pages.

Prior work suggests that perceptual organization is fast, but many of the studies involve simple stimuli with at most a small number of “groups” to detect (e.g., a single segmentation boundary). Is perceptual organization also fast in web pages, where multiple grouping cues might interact to define the menu area? To answer this question, we asked whether participants could tell where the menu bar was in a single fixation.

### Procedure

For the menu localization task, we selected from the original 714 screenshots a set of 100 screenshots not shown in any of the other experiments. Half had a menu bar along the top of the page (extending horizontally), and half had a menu bar along the left side (extending vertically). We excluded web pages that had menu bars in both locations, no menu bar, or for which the menu’s location was not obvious to two authors.

Participants performed this task interleaved with the one in Experiment 2, using the same procedure. The response screen for the menu localization task had two green buttons, labeled “side” and “top,” beneath the text, “Was the menu on the left side or the top?”

### Results

**Fig. 9:**
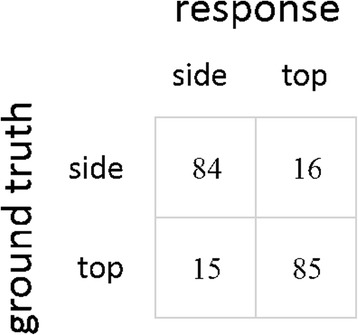
Results of menu-localization task, in percent of responses. Diagonal values indicate correct classifications

**Fig. 10:**
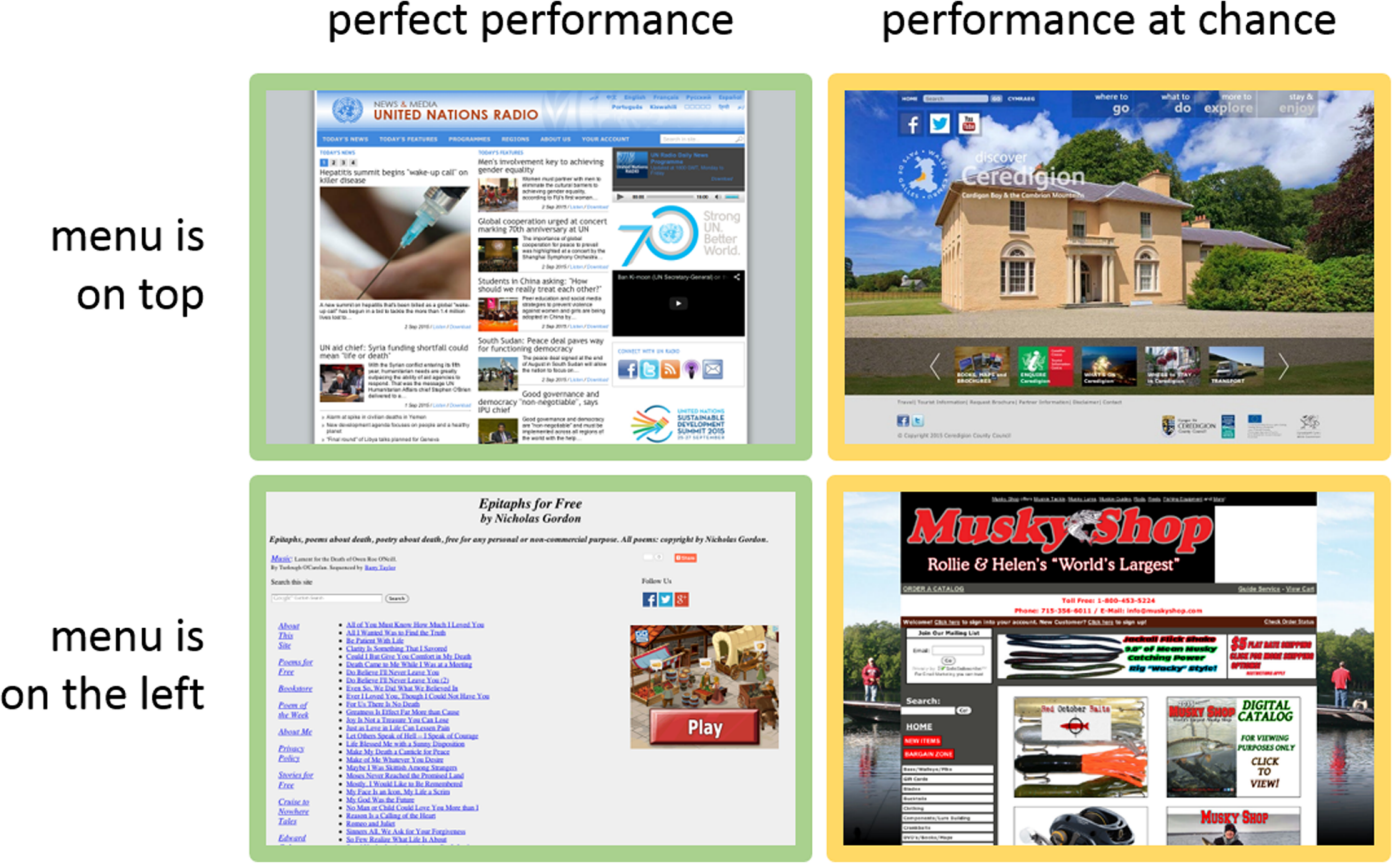
Examples of menu-localization stimuli. These example web pages contain a menu either along the top (top row) or left (bottom row), and are taken from Experiment 3, where observers had to localize the menu. The left column (green outline) shows web pages for which all 10 observers found the correct location of the menu. The second column (yellow outline) shows examples of web pages for which only half (5) of the observers were correct

Performance was high on this task (*M* = 84.6%, *SD* = 7.1), and each subject was significantly above chance (*p* < 1E-5 per subject, chance = 50%). The confusion matrix of responses is shown in Fig. 9. For example stimuli, see Fig. 10.

A single fixation provides enough information to find the menu for many designs. Given that the “hit” and “false alarm” rates for each menu location are similar, it is unlikely that the participants were biased towards responding that the menu was in either location. It is perhaps not surprising that performance on this task significantly exceeds that on the ad discrimination task (*M* = 19.9 pp., *SD* = 10 pp. over subjects, *p* < 1E-4 over all subjects and trials), as users and designers share the goal of easy page navigation, whereas their goals may be different when it comes to identifying ads.

Here, we compare some examples of web pages that had low and high performance, for both menu locations, from Experiment 3. The top left example in Fig. 10 has a grid layout grouped by color at the top, and lacks any menu-like items at the very left, suggesting a more obvious menu location. The bottom left example shows a menu clearly separated from the rest of the links. In the examples with poor performance, the menu may be confused with the other graphical elements.

## General discussion

We have demonstrated that in a single 120 ms fixation, participants can quickly categorize a web page into one of the ten common categories. Furthermore, it seems that participants are, at least in part, using text in the web pages to do the task. Future work should probe the role of text further, and ask whether participants mainly use text near the point of fixation, or whether they can read (or at least infer category from) bigger text that appears more peripherally. Clearly, however, the content of text is not the whole story; the bulk of the performance is driven by other cues, which most likely include text quantity and font, page layout, organization, and presence and content of images, among other factors. Our current work does not delineate the relative contribution of these cues, and thus a formal investigation of them is an important direction of future work. Furthermore, participants can discriminate pages that contain ads from those that do not, as well as localize elements of the layout, namely the menu bar. Ad detection is more difficult than menu localization, possibly resulting from designers’ different purposes for the two elements. Further research is needed to pin down which menu or ad styles are more visible than others, and why.

These results speak to the capabilities of the human visual system. Both recognizing scenes and recognizing web pages presumably result from similar processes that bring together extraction of a general-purpose visual representation with higher-level inference informed by previous experience. The general-purpose representation likely developed for ecologically important tasks like scene perception, but also likely supports understanding of web pages, in part because design develops to make use of existing visual processing architecture in the human visual system.

While much work has been done to study the perception of natural scenes at a glance, our study shows the importance of extending it to design research. Beyond providing the first quantitative study of rapid web page perception, we challenge previous assumptions about what can and cannot be perceived in a glance at a web page. Furthermore, researching web page perception is timely; as we become more enmeshed in the virtual world, more of our “natural vision” will be filled with artificial displays like web pages. Our expectation is that research on at-a-glance perception of digital displays and designs will become more prevalent and applicable as digital interfaces become more pervasive in daily life.

Our results also touch on practical issues. If a user clicks on a link in a web page, and the first glance or two does not suggest the correct category, she may quickly leave the page to find another one. For a well-designed page, viewing the URL at the top of the page would be far less efficient for classifying the page than getting the gist at a glance. Designers could improve existing applications by, for example, displaying text to be easily readable at a glance and suggestive of the web page’s category, or by including an easily comprehensible image that better cues the category. In working to improve their design, they could also use our technique of rapid presentation coupled with an objective categorization task to test the placement of their menus, ads, and graphical elements. While we do not test usability directly, we argue that having a quickly comprehendible web page would promote overall ease of use. This logic potentially extends to mobile devices: one can consider the first viewport of a mobile page (before scrolling) to be analogous to the first glance of a web page.

One could ask many other interesting questions about web page perception at-a-glance. What other elements can users quickly identify? How much layout information do users get at a glance? What design elements underlie the ability to accurately categorize a page, identify an ad, or find a menu? Are there computational models that can predict the results from objective tasks like ours? How does web page perception depend on the viewer’s age, experience, or visual impairment? Our hope is, beyond presenting our specific experimental results, to understand perception of designed, real-world stimuli using the rich experimental paradigms of vision science.

## References

[CR1] Baykan, E., Henzinger, M., Marian, L., & Weber, I. (2011). A Comprehensive Study of Features and Algorithms for URL-Based Topic Classification. ACM Transactions on the Web, 5(3), 15:1--15:29. 10.1145/1993053.1993057

[CR2] Benway JP (1997). Banner blindness: The irony of attention grabbing on the World Wide Web. Proceedings of the Human Factors and Ergonomics Society Annual Meeting.

[CR3] Biederman I, Kubovy M, Pomerantz J (1981). On the semantics of a glance at a scene in. Perceptual Organization.

[CR4] Buscher, G., Cutrell, E., & Morris, M. R. (2009). What do you see when you’re surfing? Using eye tracking to predict salient regions of web pages. *Proceedings of the 27th SIGCHI Conference on Human Factors in Computing Systems*, 21–30 10.1145/1518701.1518705.

[CR5] Crouzet, S. M., Kirchner, H., & Thorpe, S. J. (2010). Fast saccades toward faces: face detection in just 100 ms. Journal of Vision, 10(4), 16.1–17. 10.1167/10.4.16 10.1167/10.4.16 20465335

[CR6] Ehinger KA, Rosenholtz R (2016). A general account of peripheral encoding also predicts scene perception performance. Journal of Vision.

[CR7] Epshtein, B., Eyal, O., Yonatan, W., Ofek, E., & Wexler, Y. (2010). Detecting Text in Natural Scenes with Stroke Width Transform. *Proceedings of the IEEE Computer Society Conference on Computer Vision and Pattern Recognition, (d)*, 2963–2970 10.1109/CVPR.2010.5540041.

[CR8] Epstein R, Kanwisher N (1998). A cortical representation of the local visual environment. Nature.

[CR9] Fei-Fei L, Iyer A, Koch C, Perona P (2007). What do we perceive in a glance of a real-world scene?. Journal of Vision.

[CR10] Feigenson L, Dehaene S, Spelke E (2004). Core systems of number. Trends in Cognitive Sciences.

[CR11] Geisler WSW (2008). Visual Perception and the Statistical Properties of Natural Scenes. Annual Review of Psychology.

[CR12] Greene MR, Oliva A (2009). Recognition of natural scenes from global properties: seeing the forest without representing the trees. Cognitive Psychology.

[CR13] Greene MR, Oliva A (2009). The Briefest of Glances: The Time Course of Natural Scene Understanding. Psychological Science.

[CR14] Harris CM, Hainline L, Abramov I, Lemerise E, Camenzuli C (1988). The distribution of fixation durations in infants and naive adults. Vision Research.

[CR15] Joubert OR, Rousselet GA, Fize D, Fabre-Thorpe M (2007). Processing scene context: fast categorization and object interference. Vision Research.

[CR16] Julesz B (1981). Textons, the elements of texture perception, and their interactions. Nature.

[CR17] Kirchner H, Thorpe SJ (2006). Ultra-rapid object detection with saccadic eye movements: Visual processing speed revisited. Vision Research.

[CR18] Kleiner M, Brainard DH, Pelli DG, Broussard C, Wolf T, Niehorster D (2007). What’s new in Psychtoolbox-3?. Perception.

[CR19] Latham K, Whitaker D (1996). A comparison of word recognition and reading performance in foveal and peripheral vision. Vision Research.

[CR20] Levi D (2008). Crowding-An essential bottleneck for object recognition: A mini-review. Vision Research.

[CR21] Li FF, VanRullen R, Koch C, Perona P (2002). Rapid natural scene categorization in the near absence of attention. Proceedings of the National Academy of Sciences of the United States of America.

[CR22] Lindgaard G, Dudek C, Sen D, Sumegi L, Noonan P (2011). An exploration of relations between visual appeal, trustworthiness and perceived usability of homepages. ACM Trans. Comput.-Hum. Interact..

[CR23] Lindgaard G, Fernandes G, Dudek C, Brown J (2006). Attention web designers: You have 50 milliseconds to make a good first impression!. Behaviour & Information Technology.

[CR24] Oliva A (2005). Gist of the scene. Neurobiology of Attention.

[CR25] Oliva A, Torralba A (2001). Modeling the shape of the scene: A holistic representation of the spatial envelope. International Journal of Computer Vision.

[CR26] Oliva A, Torralba A (2006). Building the gist of a scene: The role of global image features in recognition. Progress in Brain Research.

[CR27] Palmer JW (2002). Web Site Usability, Design, and Performance Metrics. Information Systems Research.

[CR28] Pelli DG, Tillman K a (2008). The uncrowded window of object recognition. Nature Neuroscience.

[CR29] Pieters R, Wedel M (2012). Ad Gist: Ad Communication in a Single Eye Fixation. Marketing Science.

[CR30] Pirolli P, Card S (1999). Information foraging. Psychological Review.

[CR31] Potter MC (1984). Rapid Serial Visual Presentation (RSVP): A Method for Studying Language Processing. New Methods in Reading Comprehension Research.

[CR32] Rayner K, Castelhano MS, Wedel M, Pieters R (2007). Eye movements during reading, scene perception, visual search, and while looking at print advertisements.

[CR33] Reinecke, K., Arbor, A., & Gajos, K. Z. (2014). Quantifying visual preferences around the world. *Proceedings of the 32nd Annual ACM Conference on Human Factors in Computing Systems - CHI ‘14*, 11–20 10.1145/2556288.2557052.

[CR34] Rosenholtz R (2016). Capabilities and Limitations of Peripheral Vision. Annual Review of Vision Science.

[CR35] Rosenholtz, R., & Wagemans, J. (2014). *Texture Perception (pp. 1–24)*. Oxford University Press 10.1093/oxfordhb/9780199686858.013.058.

[CR36] Rousselet GA, Joubert OR, Fabre-Thorpe M (2005). How long to get to the “gist” of real-world natural scenes?. Visual Cogn.

[CR37] Shrestha S, Lenz K, Chaparro BS, Owens JW (2007). “F” Pattern Scanning of Text and Images in Web Pages. Human Factors and Ergonomics Society.

[CR38] Sperling G (1960). The information available in brief visual presentations. Psychological Monographs: General and Applied.

[CR39] Still AW, White AP (1981). The approximate randomization test as an alternative to the F test in analysis of variance. British Journal of Mathematical and Statistical Psychology.

[CR40] Thielsch MT, Hirschfeld G (2010). High and low spatial frequencies in website evaluations. Ergonomics.

[CR41] Thielsch MT, Hirschfeld G (2012). Spatial frequencies in aesthetic website evaluations – explaining how ultra-rapid evaluations are formed. Ergonomics.

[CR42] Thorpe SJ, Fize D, Marlot C (1996). Speed of processing in the human visual system. Nature.

[CR43] Thorpe SJ, Gegenfurtner KR, Fabre-Thorpe M, Bülthoff HH (2001). Detection of animals in natural images using far peripheral vision. European Journal of Neuroscience.

[CR44] Tractinsky N, Cokhavi A, Kirschenbaum M, Sharfi T (2006). Evaluating the consistency of immediate aesthetic perceptions of web pages. International Journal of Human Computer Studies.

[CR45] Treisman A (1985). Preattentive processing in vision. Computer Vision, Graphics and Image Processing.

[CR46] Treisman A, Sato S (1990). Conjunction Search Revisited. Journal of Experimental Psychology: Human Perception and Performance.

[CR47] Tullis TS (1998). A method for evaluating Web page design concepts. CHI 98 conference summary on Human factors in computing systems - CHI ‘98.

[CR48] van der Helm, P. A. (2014). In J. Wagemans (Ed.), *Simplicity in Perceptual Organization*. Oxford University Press 10.1093/oxfordhb/9780199686858.013.052.

[CR49] VanRullen R, Reddy L, Koch C (2004). Visual search and dual tasks reveal two distinct attentional resources. Journal of Cognitive Neuroscience.

[CR50] VanRullen R, Thorpe SJ (2001). The Time Course of Visual Processing: From Early Perception to Decision-Making. Journal of Cognitive Neuroscience.

[CR51] Wagemans, J. (2015). Historical and Conceptual Background: Gestalt Theory. *The Oxford Handbook of Perceptual Organization*, 3–20 10.1017/CBO9781107415324.004.

[CR52] Wedel M, Pieters R (2000). Eye Fixations on Advertisements and Memory for Brands: A Model and Findings. Marketing Science.

[CR53] Whitney, D., Haberman, J., Sweeny, T. D. (2014). From textures to crowds: multiple levels of summary statistical perception. *The New Visual Neurosciences*. MIT Press, 695–710.

